# Characterisation of pharmacogenomic variation in the Shetland and Orkney Isles in Scotland

**DOI:** 10.1038/s41598-025-26258-9

**Published:** 2025-11-26

**Authors:** David Twesigomwe, Timothy J. Aitman, James F. Wilson

**Affiliations:** 1 Sydney Brenner Institute for Molecular Bioscience, Faculty of Health Sciences, University of The Witwatersrand, Johannesburg, South Africa; 2 MRC Human Genetics Unit, Institute of Genetics and Cancer, University of Edinburgh, Edinburgh, UK; 3 Centre for Genomic and Experimental Medicine, Institute of Genetics and Cancer, University of Edinburgh, Crewe Road South, Edinburgh, EH4 2XU UK; 4 Centre for Global Health Research, Usher Institute, University of Edinburgh, Usher Building, 5-7 Little France Road, Edinburgh BioQuarter, Edinburgh, EH16 4UX UK

**Keywords:** Pharmacogenomics, Star alleles, Haplotypes, Scottish Isles, ADME, Drug response, Phenotypes, Very important pharmacogenes, Pharmacogenetics, Haplotypes, Rare variants, Structural variation, Genetic testing

## Abstract

Genetic variation is partly responsible for variability in drug response across populations. However, the full catalogue of pharmacogenetic variants and their distribution are yet to be established, thus posing challenges in implementing individualised medicine in understudied populations. This study aimed to characterise variation in key drug response genes across founder populations from the Northern Isles of Scotland. We analysed whole genome sequence datasets from 498 Shetlanders and 1372 Orcadians, the majority of whom are research participants in the Viking Genes programme, and compared the genetic variation in 41 selected pharmacogenes with observed distributions in other European datasets. From this gene-set, we present frequencies of known and potentially novel star alleles (haplotypes and structural variants) for 18 core pharmacogenes analysed using StellarPGx, and variant distributions in 23 other selected pharmacogenes with existing clinical annotations in ClinPGx (https://www.clinpgx.org). Despite important differences in the frequencies of rare and/or novel potentially high-impact variants, the distributions of the well-studied common actionable pharmacogene star alleles do not vary dramatically across Shetland, Orkney, and the European populations represented in the 1000 Genomes Project or allele frequency meta-analyses in ClinPGx. Importantly, for gene-drug pairs with Clinical Pharmacogenetics Implementation Consortium Guidelines, we estimated (based on diplotypes alone) that the proportion of participants in the combined dataset that may benefit from a change in dose/drug ranged from 0 to 50.5%, depending on the gene-drug pair. Overall, understanding the landscape of pharmacogenomic variation in Shetland and Orkney is an important step towards implementation of precision medicine across rural Scotland.

**Supplementary Information:**

The online version contains supplementary material available at 10.1038/s41598-025-26258-9.

## Introduction

Pharmacogenomics (PGx), an important facet of precision medicine, entails characterising the relationship between genetic variation across multiple genes and drug response. Several genetic variants have been associated with decreased efficacy of standard drug dosage regimens or increased risk of adverse drug reactions (ADRs) especially in genes encoding proteins involved in the absorption, distribution, metabolism, and excretion (ADME) of drugs, drug target genes, and modifier genes (such as *G6PD*, *POR*, and *VKORC1*)^1–6^. This has driven several research efforts towards supporting the implementation of genotype-guided therapy across clinical settings globally, but predominantly in Europe and North America^7–9^. Over the last decade, the feasibility of PGx has been enhanced in part due to the drop in sequencing costs and the ever-expanding technological capacity for genomic variant discovery. It is well-documented that the distribution of clinically actionable PGx variants can vary within and between populations^10–12^. Population-specific characterisation of the distribution of PGx variants or haplotypes across populations can therefore inform the prioritisation of gene-drug pairs and/or key variants for PGx testing.

Previous research has shown that isolated populations in the northern Isles of Scotland (Shetland and Orkney) tend to have genetic divergence compared to the mainland Scotland population and other populations represented in the UK Biobank, due to strong genetic drift^13,14^. Remarkably, pathogenic variants that are otherwise ultra-rare have been observed at increased frequencies in these isolates, across medically relevant genes such as *BRCA1* and *BRCA2*^15,16^. These findings are based on analysis of datasets from volunteers in the Viking Genes programme (https://viking.ed.ac.uk). This research is already informing various strategies for disease diagnosis in the Isles, complemented by other aspects such as return of genomic findings^16,17^. However, genetic variation that can impact drug response has not yet been investigated across the Viking Genes study populations. We therefore aimed to leverage the existing data from these cohorts to characterise variation in 33 Very Important Pharmacogenes (VIPs; https://www.clinpgx.org/vips) and eight other pharmacogenes with existing, though much lesser, evidence of PGx importance collated by the Pharmacogenomics Knowledge Base (PharmGKB — now integrated into ClinPGx: https://www.clinpgx.org). We hypothesised that there were likely differences in the landscape of PGx variation in Shetland and Orkney populations compared to the mainland Scotland population, and other European populations.

The 33 VIPs, in particular, have substantial evidence to support the importance of these genes in PGx^12^. VIP gene families, such as the cytochrome P450 gene family, N-acetyltransferases, glucuronidases, and solute carriers, are known to harbour considerable genetic variation, with haplotypes and/or structural variants typically represented using the star allele nomenclature^18,19^. Here we report the distribution of both clinically actionable and unannotated variation across 41 pharmacogenes in Shetland and Orkney populations, compared to the variation observed in European populations (N=503) represented in the 1000 Genomes Project datasets^20^, and those represented in the ClinPGx reference materials (aggregate data from multiple studies). Importantly, understanding the distribution of known, rare, and novel PGx variation across the Isles is relevant for PGx testing strategies with a view towards potential genetics-guided treatment optimisation interventions.

## Methods

### Ethics statement

This research is a substudy under Viking Genes (viking.ed.ac.uk), a research programme with participants from the Viking Health Study—Shetland (VIKING I) and the Orkney Complex Disease Study (ORCADES). All the VIKING I and ORCADES participants gave written informed consent for broad ranging health and ancestry research including whole genome/exome sequencing.

VIKING I was approved by the South East Scotland Research Ethics Committee (REC Ref 12/SS/0151). ORCADES was given a favourable opinion by Research Ethics Committees in Orkney, Aberdeen (North of Scotland REC), and South East Scotland REC, NHS Lothian (reference: 12/SS/0151). Both cohorts are now part of Viking Genes, with a favourable opinion from NHS Lothian South East Scotland Research Ethics Committee (reference 19/SS/0104).

All methods were carried out in accordance with relevant guidelines and regulations.

### Study participants

**Fig. 1:**
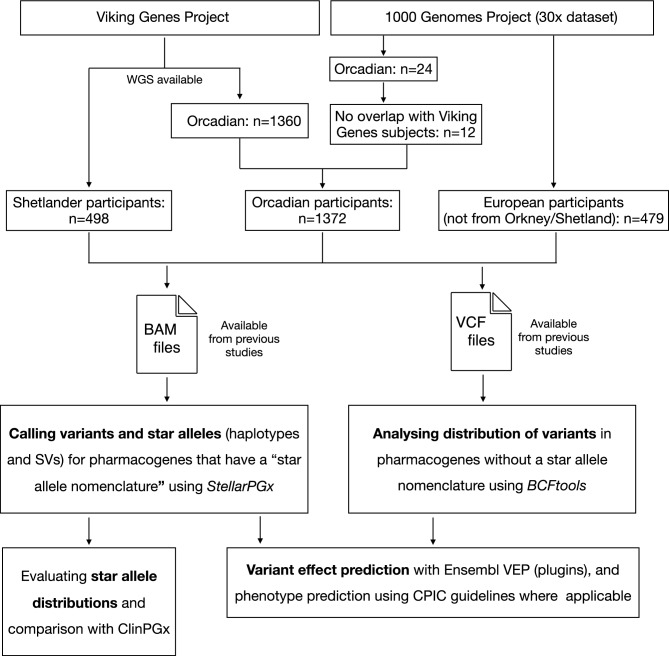
Overview of the study design and outline. Viking Genes is a project including cross-sectional studies aiming to identify genetic factors influencing complex disease risk in the population isolates of the Shetland and Orkney Isles in northern Scotland. Binary Alignment Map (BAM) files and Variant Call Format (VCF) files used in this study have been generated in previous studies in Viking Genes^14^ and by the New York Genome Center (for the 1000 Genomes datasets)^20^. SV: Structural Variant, VEP: Variant Effect Predictor, CPIC: Clinical Pharmacogenetics Implementation Consortium.

Figure 1 provides a general overview of the study outline. The study included participants from the Viking Health Study - Shetland (VIKING I)^17^ and the Orkney Complex Disease Study (ORCADES)^21^, which are family-based, cross-sectional studies aiming to identify genetic factors influencing complex disease risk in the population isolates of the Shetland and Orkney Isles in northern Scotland. These studies, along with VIKING II, are part of the Viking Genes programme (https://www.ed.ac.uk/viking). A detailed description of Viking Genes has been published in previous work^13,14,17,21^. For the pharmacogenomics analysis in this study, we included individuals for whom whole genome sequence (WGS) data is available, i.e. 498 participants from the Shetland archipelago via the Scottish Genomes Partnership^22^ and 1360 from Orkney sequenced at the Wellcome Sanger Institute^23^. From a WGS perspective, the average read depth across the Shetland WGS data was >30x, while the read depth across the Orkney WGS datasets was generally lower (interquartile range 15-20x). For allele frequency comparisons with other European populations, we recalled star alleles from the 30x WGS data of the 503 European-ancestry participants in the 1000 Genomes Project (1000G European participants)^20^, and we also used allele frequency data aggregated by ClinPGx from several previous studies (https://www.clinpgx.org/page/pgxGeneRef). Of the 1000G European participants, 24 are Orcadian; therefore, we removed them from the 1000G European group—remaining with 479 participants. Of the 24 Orcadian participants, 12 were already overlapping with the existing Orkney WGS dataset—we added the other 12 to this dataset to bring the total number of Orcadian participants in the study to 1372.

### Variant and star allele calling

Our analysis was largely focused on 33 genes categorised by ClinPGx (formerly PharmGKB) as VIPs (including *ACE*, *ABCB1*, *ABCG2, ADRB1*, *ADRB2, CACNA1S*, *CFTR, COMT, CYP2A6, CYP2B6*, *CYP2C19*, *CYP2C9*, *CYP2C8*, *CYP2D6, CYP3A4*, *CYP3A5*, *CYP4F2*, *DRD2, DPYD*, *G6PD, GSTM1*, *GSTP1, IFNL3, MTHFR, NAT2, NUDT15*, *RYR1, SLCO1B1*, *SLC19A1, TPMT*, *TYMS, UGT1A1* and *VKORC1*). Secondary to these genes, we analysed 6 drug metabolism and transporter genes (i.e. *CYP1A1, CYP1A2, CYP2E1, GSTT1*, *SLC22A1* and *SULT1A1*) that are included in the ClinPGx’s former Tier 2 VIP list^24^. We also included *POR* owing to its importance in the functioning of CYP genes^25^, and *NAT1* given its proximity to *NAT2* and involvement in the metabolism of cotinine. Thus, in total we analysed 41 pharmacogenes. For genes without a standardised star allele nomenclature (*ABCB1*, *ABCG2, ACE*, *ADRB1*, *ADRB2, CACNA1S*, *CFTR, COMT, CYP1A1*, *CYP2E1*, *DPYD*, *DRD2, G6PD, GSTP1*, *IFNL3*, *MTHFR, RYR1, SLC19A1, SLC22A1, SULT1A1, TYMS* and *VKORC1*), we subset existing WGS variant call format (VCF) files generated from previous joint-calling analyses on Viking Genes datasets performed using the Genome Analysis Toolkit^14,26^. VCF files for the 1000G European participants were obtained from ftp.1000genomes.ebi.ac.uk. For this gene-set, we proceeded directly to the variant annotation steps for the passed SNVs (see below). For genes with a star allele nomenclature (*CYP1A2*, *CYP2A6*, *CYP2B6, CYP2C19*, *CYP2C9*, *CYP2C8*, *CYP2D6, CYP3A4*, *CYP3A5*, *CYP4F2*, *GSTM1, GSTT1, NAT1*, *NAT2, NUDT15*, *POR, SLCO1B1, TPMT*, *UGT1A1*), we used StellarPGx v1.2.8 to determine the corresponding diplotypes for each individual^27^. StellarPGx (https://github.com/SBIMB/StellarPGx) takes high coverage (≥20x) WGS Binary Alignment Map (BAM) files as input. The pipeline detects single nucleotide variations and indels (herein collectively referred to as SNVs) using GraphTyper, a pangenome-based variant caller^28^. Following variant detection, StellarPGx retains only variants with quality by depth (QD) >10, and allele balance for heterozygous calls (ABHet) between 0.25 and 0.75 (inclusive), to improve accuracy in the subsequent star allele assignment steps. StellarPGx assigns candidate star alleles, based on core (allele-defining) variant combinations in the VCFs, and then combines this information with copy number changes (if detected) based on read-depth output from SAMtools^29^ to produce final pharmacogene diplotype calls. Core variants across VIPs typically include missense variants, frameshift variants, stop gain/loss variants, regulatory variants and/or variants resulting in splice defects (https://www.pharmvar.org/criteria). StellarPGx is optimised to detect and phase duplications, and other structural variants, based on read-depth distributions and variant allele depth ratios.

The star allele definitions in the StellarPGx database are based on definitions provided by the Pharmacogene Variation (PharmVar) Consortium database (https://www.pharmvar.org; last accessed March 18, 2025), the UGT nomenclature database (https://www.pharmacogenomics.pha.ulaval.ca/ugt-alleles-nomenclature/), and the *TPMT* nomenclature committee (https://liu.se/en/research/tpmt-nomenclature-committee). For individuals with novel star alleles, StellarPGx includes a flag in the output indicating the presence of a potential novel allele (this would mean that none of the diplotypes in the StellarPGx database is a match) and recommends extra manual variant inspection and experimental validation. Novel star alleles could be due to occurrence of novel function-altering variants on common haplotypes (‘backbones’) or novel combinations of existing core variants defining other haplotypes in the pharmacogene of interest. In this study, ambiguous diplotype calls that were not due to occurrence of potentially novel star alleles or structural variants were resolved using phasing information from SHAPEIT4^30^.

### Variant annotation and phenotype predictions

All the core variants across the VIPs in this study were annotated using the Ensembl variant effect predictor (VEP)^31^. VEP plugins including SIFT^32^, Polyphen-2^33^, CADD^34^, LRT^34^, PROVEAN^35^, MutationAssessor^36^, and AlphaMissense^37^ were used to predict potential variant deleteriousness. Phenotype predictions for selected VIPs were obtained from diplotype information via published criteria by the Clinical Pharmacogenetics Consortium (CPIC; https://cpicpgx.org/guidelines), where applicable.

### Statistical analysis

The Fisher’s exact test was used to determine any significant differences in star allele frequencies between different populations. For correction of multiple-testing correction, we applied the false discovery rate p-value adjustment for dependent observations^38^. In these analyses, p-values < 0.05 indicated a significant difference. Possible deviations from Hardy–Weinberg equilibrium (HWE) were investigated using the Genetics package (v1.3.8.1.3) in R v4.1.0 (www.r-project.org/).

## Results

The average read depth across the 41 pharmacogenes analysed in the study was 35x and 20x across Shetland and Orkney WGS datasets, respectively (Figure S1). As expected, the X-linked *G6PD*, and commonly deleted *GSTM1* and *GSTT1* were outliers in this regard. Across the 41 pharmacogenes, there were 30,539 variants combined across the two datasets. About two-thirds (n=20,334) of these variants were rare (MAF<1%) in both the Shetland and Orkney datasets, with 39% (n=11,922) being singletons in either population. Over 6.7% (n=2038) of the total variants were novel (i.e. not in the database of single nucleotide polymorphisms; dbSNP v154).

### Variation in Very Important Pharmacogenes highlighted by CPIC guidelines

**Fig. 2:**
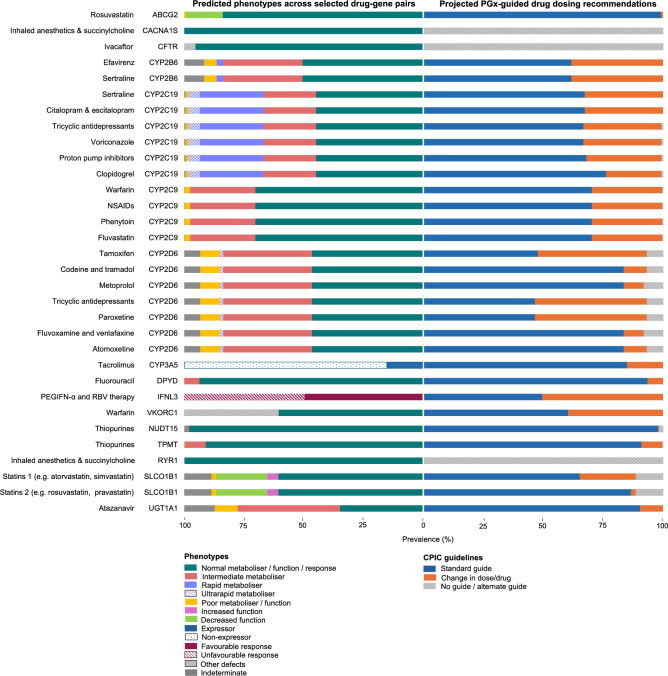
Predicted pharmacogene phenotype variability (left) across Orcadian and Shetlander study populations in Viking Genes and the proportion of participants that may benefit from genomics-informed drug therapy (right). The phenotypes were predicted from variants and/or star alleles called in the study based on published guidelines by the Clinical Pharmacogenetics Consortium (CPIC; https://cpicpgx.org/guidelines). The projected PGx-guided drug dosing recommendations were also based on CPIC guidelines. For example, for ABCG2, a combined 0.8% of the study participants in Orkney and Shetland were predicted to have poor ABCG2 function, while 15.4% were predicted to have decreased ABCG2 function. The CPIC guideline for ABCG2 and rosuvastatin recommends a reduced starting dose of ≤20 mg (and adjustment based on disease-specific and population-specific guidelines) for the individuals with poor ABCG2 function, but not for the individuals with decreased ABCG2 function. Therefore, while the left half of the figure is intended to highlight the variability in the predicted phenotype proportions in Shetland and Orkney, the right half of the figure is meant to summarise what proportions of individuals (per gene-drug pair) are projected to potentially require change in drug/dose if pharmacogenetic testing and CPIC guidelines were to be leveraged.

Across the Shetland and Orkney datasets, all the participants (100%) had at least one clinically actionable variant and/or star allele combination (diplotype), across 18 of the VIPs with existing Clinical Pharmacogenetic Implementation Consortium (CPIC) guidelines. The number of these genotypes per individual varied between 2 and 10 (median=6; mean=5.8) in the Shetland population, while it varied between 2 and 11 (median=6; mean=5.6) in the Orcadian dataset. We observed a comparable distribution in the 1000G European dataset (median=6; mean=5.9; range of 2 to 11 clinically actionable diplotypes). Across gene-drug pairs with CPIC guidelines, the proportion of Shetlanders and Orcadians predicted (based on diplotypes alone) to potentially benefit from pharmacogenetics-guided treatment interventions ranged from 0 to 50.5% (mean = 22.2%), depending on the gene-drug pair. The actionable phenotypes occurred predominantly in CYP2B6, CYP2C19, CYP2C9, CYP2D6, IFNL3, SLCO1B1, and VKORC1 (Figure 2 and Table S1). Overall, there were no significant differences in the predicted phenotype distributions between the Shetland, Orkney, and the 1000G European study populations (Table S1).

In *CYP2B6*, we identified 15 star alleles in the Shetland and Orkney datasets. CYP2B6*6 (decreased function) was the most common clinically actionable allele at a frequency of 17.3% and 16%, respectively. There were no significant differences in the *6 frequency when compared with other European populations (Table 1). Other *CYP2B6* decreased function alleles with AF>1% were *7 and *9. CYP2B6*4 and *22 (increased function alleles) were relatively rare (Table 1). CYP2B6*36 (decreased function) was observed in the Shetland dataset (AF=0.4%), but it was absent in the Orkney dataset.

**Table 1:** Common star alleles in selected cytochrome P450 genes across Shetland and Orkney cohorts in comparison with other European populations. Rare alleles in these genes are presented in Supplementary table 2.

Star alleles	Defining variants	CPIC/known clinical function	Frequencies (%)			
			Shetland	Orkney	European (1000G)	European (ClinPGx)^b^
CYP2A6						
*1	Reference haplotype	Normal enzyme activity	48.8	44.1	51.3	n/a
*2	rs1801272 (L160H)	Substantially decreased enzyme activity	3.7	3.0	3.2	n/a
*9	rs28399433 (TATA Box)	Decreased mRNA expression	6	8.1	7.0	n/a
*14	rs28399435 (S29N)	Unknown	2.8	2.3	3.2	n/a
*18	rs1809810 (Y392F)	Decreased enzyme activity	1.1	2.2	1.4	n/a
*21	rs6413474 (K476R)	Decreased enzyme activity	0.8	1.9	1.2	n/a
*35	rs143731390 (N438Y), 3’-UTR conversion (expression)	Decreased enzyme activity	5.8	2.6	0.8	n/a
*46	3’-UTR conversion (expression)	Increased mRNA stability	27.7	34.2	27.2	n/a
CYP2B6						
*1	Reference haplotype	Normal	51.1	54.8	53.4	49.1
*2	rs8192709 (R22C)	Normal	6.4	5.3	5.2	4.9
*4	rs2279343 (K262R)	Increased	1.7	1.0	3.1	4.1
*5	rs3211371 (R487C)	Normal	12.5	10.7	10.8	11.5
*6	rs3745274 (Q172H, splice defect), rs2279343 (K262R)	Decreased	17.3	16	22.1	23.3
*7	rs3745274 (Q172H, splice defect), rs2279343 (K262R), rs3211371 (R487C)	Decreased	3.1	2.9	0	2.5
*9	rs3745274 (Q172H, splice defect)	Decreased	2.0	3.8	0.6	1.5
*11	rs35303484 (M46V)	Uncertain	1.1	1.0	0.4	0.3
*15	rs35979566 (I391N)	Uncertain	0.9	1.6	0.4	0.7
*22	rs34223104 (expression)	Increased	1.9	0.9	0.9	1.4
CYP2C19						
*1	rs3758581 (I331V)	Normal	59.6	59.8	54.8	62.5
*2	rs12769205 (splicing defect), rs4244285 (splicing defect), rs3758581 (I331V)	No function	13.3	11.0	14.8	14.7
*17	rs12248560 (expression), rs3758581 (I331V)	Increased	20.3	20.5	22.5	21.5
*38	Reference haplotype	Normal	5.6	7.0	6.8	
CYP2C9						
*1	Reference haplotype	Normal	81.4	84.3	78.7	79.3
*2	rs1799853 (R144C)	Decreased	12	7.9	12.9	12.7
*3	rs1057910 (I359L)	No function	5.6	7.4	7.3	7.6
CYP2C8						
*1	Reference haplotype	Normal	79.4	87.8	81.6	n/a
*3	rs11572080 (R139K), rs10509681 (K399R)	Uncertain	12.5	7.7	12.3	n/a
*4	rs1058930 (I264M)	Uncertain	5.8	4.1	5.6	n/a
*15	rs41286886 (V181I)	Uncertain	2.1	0	0	n/a
CYP2D6						
*1	Reference haplotype	Normal	33	36.8	35.9	28.5
*2	rs16947 (R296C), rs1135840 (S486T)	Normal	14.2	13.7	15.7	18.5
*2x2	Duplication of *2	Increased	0.4	0.3	1.7	1.2
*3	rs35742686 (frameshift)	No function	1.5	1.6	1.3	1.6
*4	rs3892097 (splicing defect)	No function	17.6	15.9	11.3	18.5
*4xN	Duplication of *4	No function	2	2.1	1.1	0.7
*68+*4	CYP2D6-2D7 hybrid (intron 1 switch point) in tandem with *4	No function	2.8	2.5	5.9	
*5	Full gene deletion	No function	3.5	4.4	2.4	2.9
*6	rs5030655 (frameshift)	No function	0.9	1.3	2.2	1.1
*9	rs5030656 (K281del)	Decreased	4.8	4.7	2.4	2.8
*10	rs1065852 (P34S), rs1135840 (S486T)	Decreased	0.3	0.8	1.3	1.6
*28	rs72549358 (V7M), rs78482768 (Q151E), rs16947 (R296C), rs1135840 (S486T)	Uncertain	0.8	1	0.5	0.4
*33	rs28371717 (A237S)	Normal	1.3	0.4	0.6	1.0
*35	rs769258 (V11M), rs16947 (R296C), rs1135840 (S486T)	Normal	8	4.5	5	5.5
*41	rs16947 (R296C), rs28371725 (splicing defect), rs1135840 (S486T)	Decreased	4.9	4.8	8.4	9.2
*59	rs16947 (R296C), rs79292917 (splicing defect), rs1135840 (S486T)	Decreased	1	0.8	0.2	0.4
CYP2E1^b^						
*4	rs6413419-A (V179I)	Uncertain	1.4	2.2	3.2	n/a
*1x2	Gene duplication	Uncertain	1.1	1.4	1.0	n/a
CYP3A4						
*1	Reference haplotype	Normal	93.6	92	93.7	n/a
*3	rs11572080 (R139K), rs10509681 (K399R)	Uncertain	0.5	1.2	0.7	n/a
*10	rs1058930 (I264M)	Uncertain	0.6	1.3	0.3	n/a
*22	rs41286886 (V181I)	Uncertain	4.9	5.4	4.5	n/a
CYP3A5						
*1	Reference haplotype	Normal	3.5	9.9	4.9	7.4
*3	rs776746 (splice defect)	No function	96.5	90.1	94.8	92.4
CYP4F2						
*1	Reference haplotype	Normal	61	58.1	57.5	56.6
*3	rs2108622 (V433M)	Unknown	11.1	15.4	11.3	27.5
*4	rs2108622 (V433M), rs3093105 (W12G)	Unknown	14.3	12.4	17.6	
*5	rs3093200 (L519M)	Unknown	6.3	6.7	5.9	
*6	rs3093153 (G185V)	Unknown	4.6	5	6.7	
*14	rs145174239 (L341V)	Unknown	1.6	1.5	0.3	

^a^CYP2E1 star allele nomenclature has not been fully curated/updated since 2013.

^b^Number of participants in ClinPGx aggregate analyses vary (CYP2B6: n=69572, CYP2C19: n=71782, CYP2C9: n≈91200, CYP2D6: n=65090, CYP3A5: n=5607, CYP4F2: n=77524).

In *CYP2C19*, we observed 8 star alleles across the Shetland and Orkney datasets. CYP2C19*2 (no function) and *17 (increased) were the most frequently observed clinically actionable *CYP2C19* alleles and their frequencies were similar to the distribution in other European populations (Table 1).

In *CYP2C9*, we observed 7 star alleles across the Shetland and Orkney datasets. The frequency of CYP2C9*2 (decreased function) was slightly higher in the Shetland population (AF=12%) than in Orkney (~8%). Conversely, the frequency of CYP2C9*3 was slightly lower in Shetland (AF=5.6%) than in the Orkney population (AF=7.4%). However, these differences were not statistically significant and the distributions in Shetland were similar to observations among other European populations (Table 1). CYP2C9*11 and *12 were present in both datasets but observably rare (Table S2). Among other genes important for warfarin response, we estimated about 60% of Shetlander and Orcadian participants to have the VKORC1 warfarin low dose phenotype, with 13–14% being homozygous for the clinically actionable rs9923231-T variant (–1639G>A). The frequency of rs9923231-T was similar in both populations (36–37%) and to the frequency in other European populations (Table S3). In *CYP4F2*, the frequencies of the *3 and *4 alleles (both of which contain the ClinPGx level 1 A rs2108622 variant) were estimated to range between 11–15.5.5% across the Shetland and Orkney populations.

In *CYP2D6*, we called 37 individual star alleles across the Shetland and Orkney datasets. The allele frequency of CYP2D6*2 (normal function) was estimated to be in the range of 13% to 15%. The frequency of the non-functional CYP2D6*4 allele (defined by rs3892097; splice defect) was observed to be around 17.5% and 16% in Shetland and Orkney (slightly higher than AF in the 1000G European participants i.e. 11.3%), respectively, while the frequency of the CYP2D6*68+*4 hybrid tandem arrangement was estimated to be 2.5–3.5% (lower than in 1000G European participants i.e. around 6%). The ClinPGx resource reported a CYP2D6*4 frequency of 18.5% for the European biogeographical group, but did not have a recorded frequency for *68+*4 at the time of this study. CYP2D6*5 (full gene deletion), *9 (decreased function), and *41 (decreased function) were observed at frequencies of 3.5–5%. Despite observably lower allele frequencies for both *5 and *9, and higher frequencies for *41, in other European populations (Table 1), these differences were not statistically significant. Duplication alleles including CYP2D6*1x2, *2x2, *3x2, and *35x2 were found to be relatively rare (AF≤0.5%) in the Shetland and Orkney populations (Table 1).

In *CYP3A5*, the *3 allele (splice defect) was observed at frequencies >90% in both Shetland and Orkney. Around 15% of the participants were predicted to be CYP3A5 expressors, but the proportion was significantly higher in the Orkney participants (17.9%) than among the Shetland participants (7%) (p<0.01). In comparison, the proportion of CYP3A5 expressors predicted among the 1000G European participants was 9.4%, and the proportion estimated in ClinPGx among European participants is about 14.2%.

Approximately 6.5% of the Shetland and Orkney participants were predicted to be DPYD intermediate metabolisers. The most common clinically actionable *DPYD* variations observed in these cohorts were the ‘HapB3’ haplotype variants i.e. rs56038477 (E412E); rs75017182 (splice defect) (frequency of 2.7% and 2.9%, in Shetland and Orkney, respectively). Other known high impact variants observed were rs67376798 (D949V) and rs3918290 (splice defect).

For genes relevant to thiopurine response, we estimated about 8.6% of Shetland and Orkney participants to be TPMT intermediate metabolisers while only 0.4% of participants were NUDT15 intermediate metabolisers (Table S1). The most prevalent alleles contributing to these phenotypes were TPMT*3A (AF of 3.8% and 3.2% in Shetland and Orkney, respectively) and NUDT15*3 (AF of 0.4% and 0.1% in Shetland and Orkney, respectively). The frequency of TPMT*3A in the 1000G European participants (1.6%) was significantly lower than that in Shetland (p=0.002) and Orkney (p=0.005); however, the *3A frequency in other European populations (ClinPGx) was similar to that in Shetland and Orkney. There was no data for *3E in ClinPGx. The frequencies for all *TPMT* and *NUDT15* star alleles in the datasets are provided in Table 2.

**Table 2:** Star alleles in selected non-cytochrome P450 pharmacogenes across Shetland and Orkney cohorts in comparison with other European populations.

Star alleles	Defining variants	CPIC/Known clinical function^a^	Frequencies (%)			
			Shetland	Orkney	1000G European datasets	European (ClinPGx)^b^
GSTM1						
*A	Reference haplotype	Normal	18.5	13.0	18.2	n/a
*B	rs1065411 (K173N)	Normal	7.9	7.7	8.1	n/a
*0	GSTM1 deletion	No function	72.7	77.6	71.6	n/a
*Ax2	Duplication of *A	Increased activity	0	0	0.2	n/a
*Bx2	Duplication of *B	Increased activity	0.2	<0.1	0	n/a
GSTT1						
*A	Reference haplotype	Normal	42.4	42.5	59.3	n/a
*0	GSTT1 deletion	No function	57.4	57.5	39.9	n/a
*Ax2	GSTT1 duplication	Increased activity	0	0	0.2	n/a
NAT1						
*3	rs15561-A	Uncertain	3.2	3.9	2.0	n/a
*4	rs15561-C, rs1057126-T	Normal	75.0	73.8	69.4	n/a
*10	rs15561-A, rs1057126-A	Increased activity	16.0	18.8	21.8	n/a
*11 (including *11A, 11B and *11C)	rs4987076 (V149I), rs4986783 (S214A), rs4986990 (T153T), rs72554666 (delAATAATAAA), rs15561-A	Increased activity	1.8	1.5	2.3	n/a
*14A and *14B	rs4986782 (R187Q), rs15561-A, rs1057126-A	Decreased activity	2.4	1.0	2.1	n/a
*15	rs5030839 (R187*)	No function	0.4	0.2	0.9	n/a
*17	rs56379106 (R64W)	Decreased activity	0.4	0.5	0.5	n/a
*18A	rs4646271-delAAA, rs1057126-A, rs15561-A	Uncertain	0	0.1	0.4	n/a
*22	rs56172717 (D251V)	Decreased activity	0	0	0.5	n/a
*29	rs15561-A, rs1057126-A, rs8190859	Uncertain	0.8	0.1	0	n/a
NAT2						
*1	Reference haplotype	Rapid acetylation	0.3	1.2	0.4	n/a
*4	rs1208 (R268K)	Rapid acetylation	6.5	19.5	24.2	n/a
*5	rs1801280 (I114T)	Slow acetylation	40.9	39.2	43.2	n/a
*6	rs1799930 (R197Q), rs1208 (R268K)	Slow acetylation	41.4	33.9	30.0	n/a
*7	rs1208 (R268K), rs1799931 (G286E)	Slow acetylation	3.1	2.1	2.4	n/a
*14	rs1801279 (R64Q), rs1208 (R268K)	Slow acetylation	0	0	0.1	n/a
*16	rs1801280 (I114T), rs1208 (R268K)	Slow acetylation	5.7	3.5	1.7	n/a
*30	rs1801280 (I114T), rs1799930 (rs1799930), rs1208 (R268K)	Slow acetylation	0	<0.1	0	n/a
*34	rs1799930 (R197Q)	Slow acetylation	0	0.4	0	n/a
*40	rs1799931 (G286E)	Unknown	0	<0.1	0	n/a
NUDT15						
*1	Reference haplotype	Normal	98.2	99.3	99.8	99.3
*3	rs116855232 (R139C)	No function	0.4	0.1	0.2	0.2
*4	rs147390019 (R139H)	Unknown	0	<0.1	0	<0.1
*12	rs149436418 (F52L)	Unknown	1.4	0.5	0	
SLCO1B1						
*1	Reference haplotype	Normal	57.8	59.6	56.6	40.3
*4	rs11045819 (P155T)	Unknown	0	0.2	0.1	
*5	rs4149056 (V174A)	No function	2.9	2.7	2.7	2.0
*14	rs2306283 (N130D), rs11045819 (P155T)	Increased	12.9	15.8	14.1	13.6
*15	rs2306283 (N130D), rs4149056 (V174A)	No function	11.4	11.4	13.0	15.0
*19	rs34671512 (L643F)	Unknown	0.4	0.8	0.3	
*20	rs2306283 (N130D), rs34671512 (L643F)	Increased	7.5	5.6	4.8	3.7
*37	rs2306283 (N130D)	Normal	7.0	3.9	8.0	25.3
*40	rs4149056 (V174A), rs34671512 (L643F)	Uncertain	0	<0.1	0	
*46	rs2306283 (N130D), rs4149056 (V174A), rs71581941 (R580X)	No function	0	0	0.2	
*48	SLCO1B1 gene deletion	No function	0	<0.1	0	
TPMT						
*1	Reference haplotype	Normal	95.6 (*1S=21.9)	95.4 (*1S=21.2)	95.9 (*1S=20.9)	95.3
*2	rs1800462 (Ala80Pro)	No function	0.2	0.1	0.6	0.2
*3A	rs1800460 (Ala154Thr), rs1142345 (Tyr240Cys)	No function	3.8	3.2	1.6	3.4
*3B	rs1800460 (Ala154Thr)	No function	0	0.04	0	0.3
*3C	rs1142345 (Tyr240Cys)	No function	0.3	0.1	0.1	0.5
*3E	rs1800460 (Ala154Thr), rs2842934 (Ile158Ile), rs1142345 (Tyr240Cys)	No function	0	0.9	1.1	
*9	rs151149760 (Lys119Thr)	Uncertain	0.1	0.1	0	<0.1
*12	rs200220210 (S125L)	Uncertain	0	0	0.1	
*16	rs144041067 (R163P)	Uncertain	0	0	0.2	0.1
*21	rs200591577 (L69V)	Uncertain	0	0	0.1	<0.1
*32	rs115106679 (E114K)	Uncertain	0	0	0.1	<0.1
*33	rs112339338 (R163C)	Uncertain	0	0	0.1	<0.1
UGT1A1						
			Shetland	Orkney	1000G European datasets	European (ClinPGx), n=1550
*1	Reference haplotype	Normal	50.5	58.6	56.4	36.1
*6	rs4148323 (G71R)	Decreased function	0	0	0.6	0.8
*28	rs3064744 (A[TA]6TAA>A[TA]7TAA; expression)	Decreased function	0	0.4	0.3	31.6^b^
*28+*80	rs887829 + rs3064744 (A[TA]6TAA>A[TA]7TAA; expression)	Decreased function	32.8	23.6	27.6	
*80	rs887829	Unknown	0.3	1.1	0	
*36	rs3064744-delTA	Increased function	0	0.3	0.3	0.0
*37+*80	rs887829 + rs3064744 (A[TA]6TAA>A[TA]8TAA; expression)	Unknown	0.3	0	0	
*65	rs28899472	Unknown	0.5	0.2	0.5	
*66	rs2302538	Unknown	11.3	12.6	11.8	

^a^Functional impacts of alleles in GST genes, NAT genes, TPMT, UGT1A1 were obtained from published literature.

+Number of participants in ClinPGx aggregate analyses vary (NUDT15: n≈52267, SLCO1B1: n≈60448, TPMT: n≈95315, UGT1A1: n=1550).

^c^The UGT1A1*28 frequency in the ClinPGx reference materials is reported as ~31.6% and that for UGT1A1*80 is reported as ~31.4%, but the *28 allele is known to be in high linkage disequilibrium with *80.

Across drug transporter genes with existing CPIC guidelines (*SLCO1B1* and *ABCG2*), we estimated about 21.4% of the Shetland and Orkney participants to have *SLCO1B1* (OATP1B1 encoding-gene) decreased function and about 4.7% of the participants to have OATP1B1 increased function. The common *SLCO1B1* alleles contributing to the decreased function phenotype included *15 (no function) followed by *2 (no function) (Table 1). SLCO1B1*14 and *20 were the predominant increased function star alleles. The SLCO1B1*20 frequency was relatively elevated in the Shetland population (7.5%) compared to the 1000G European populations (4.8%; p=0.536) and to the data from studies curated by ClinPGx (3.7%; p<0.01) (Table 1). In *ABCG2*, the frequency of rs2231142 (Q141K) was estimated to be 10.2% and 7.9% in Shetland and Orkney, respectively (Table S3). We predicted 15.4% of the Shetland and Orkney participants to have decreased ABCG2 function, while 0.8% were predicted to have poor ABCG2 function.

In *UGT1A1*, we predicted 43% of the Shetland and Orkney participants to be intermediate metabolisers and 9.6% to be poor metabolisers. The key UGT1A1*28 allele was found in combination with *80 (i.e. as UGT1A1*28+*80) in nearly every occurrence (Table 2). The second most prevalent non-reference *UGT1A1* allele was *66 (AF=11.3% and 12.6% in Shetland and Orkney, respectively).

Across other genes with existing CPIC guidelines (Table S3); for *CFTR*, rs199826652 (F508del-*CFTR*) was the more common (average AF=1.7%) of the actionable variants, while rs75527207 (G551D) was rare (AF=0.05%). For IFNL3, around 50% of the participants were predicted to have unfavourable response with regard to Pegylated interferon-α and ribavarin therapy. The average frequency of the *IFNL3* rs12979860-T allele was estimated at around 30.2%. In *RYR1*, only two participants from Shetland had a known clinically actionable variant (rs892052), while no known actionable variants were observed in *CACNA1S*. In *G6PD*, only a small subset of Shetlander (2%) and Orcadian (1%) participants, had non-synonymous variant(s). All the non-synonymous *G6PD* variants were rare, except rs370017540-T (G346D) which, although rare in Orkney (AF<0.1%), was relatively more frequent in Shetland (AF=0.8%).

### Variation in VIPs with recently updated star allele nomenclature

In *CYP2A6*, we called 15 star alleles in Shetland and Orkney combined. Besides CYP2A6*1, CYP2A6*46 (3’-UTR conversion) was the most common allele at a frequency of 27% in Shetland and 34% in Orkney (Table 1). Other *CYP2A6* structural variants, including *1x2, *4 (gene deletion), *12 (exons 1–2 from *CYP2A7*), and *47 (exons 1–8 from *CYP2A7*) were observably rare (Table S2). Among the star alleles associated with decreased CYP2A6 activity or mRNA expression, CYP2A6*2, *9 and *35 were relatively common but occurred at frequencies <10%.

In *NAT2*, *1 and *4 (rs1208, R268K) were the only rapid acetylation alleles observed across Shetland and Orkney participants. Among the *NAT2* slow acetylation alleles, *5 (rs1801280, I114T), *6 (rs1799930, R197Q; rs1208, R268K), and *16 (rs1801280, I114T; rs1208, R268K) were the most common (Table 2).

### Predicted high impact SNVs among variants lacking validated clinical functional annotation

Across VIPs with existing CPIC guidelines, we predicted 176 variants to have potentially high (deleterious) impact on the respective protein, based on the consensus of VEP plugins used in the study (Table S3). Frameshifts, stop gain variants, and splice defects were considered deleterious by default. Among the variants without current CPIC functional annotation, 12 including *ACE* rs3730025-G (Y244C); *CACNA1S* rs12139527-G (L1800S), rs3850625-A (R1539C) and rs12406479-C (A69G); *CFTR* rs1800076-A (R75Q), rs1800098-C (G576A) and rs1800100-T (R668C); *CYP2B6* rs35979566-A (I391N); *CYP3A4* rs4986910-G (M445T); *MTHFR* rs1801133-A (A222V) and rs142617551-A (E470V); and *NUDT15* rs149436418-G (F52L) were common (AF≥1%) across Shetland and/or Orkney, while the majority were relatively rare (Table S3).

Across pharmacogenes without current CPIC guidelines, 74 variants were predicted to be deleterious by the consensus of VEPs used (frameshifts, stop gain variants, and splice defects were considered deleterious by default). Of these, 11 variants including *ABCB1* rs2032582-T (S893T), *CYP2A6* rs1801272-T (*2-defining variant; L160H), *CYP2E1* rs6413419-A (V179I), *DRD2* rs1801028-C (S311C), rs71653614-C (K327E), *GSTM1* (N85S), *SLC22A1* rs12208357-T (R61C), rs34059508-A (G465R), rs2282143-T (P341L), rs34130495-A (G401S), and *SULT1A1* rs376524943-G (G259A) were common (AF≥1%) in Shetland and/or Orkney, while others were relatively rare (Table S3).

### Potentially novel star alleles in Shetland and Orkney inferred from WGS data

Across the genes in the current PharmVar database (v6.2.2), we observed a combined total of 46 potentially novel star alleles in *CYP2D6*, *CYP2B6*, *CYP2A6*, *CYP2C19*, *CYP2C9*, *CYP2C8*, *CYP3A4*, and *CYP4F2* (Table S4). The most common of these haplotypes included: *9+rs145308399~5576G>A(E97K) in *CYP2A6* and *1+rs138264188~17731C>T (T67M) in *CYP2B6*, especially among Orcadians (AF=0.4% and 0.7%, respectively). We also identified 8 potentially novel suballeles comprising nonsynonymous variants on non-functional backbones in *CYP2D6*, *CYP2C19* and *CYP3A5* (Table S4)*.* The more common of these suballeles was *2+rs58973490~17827G>A(R150H) in *CYP2C19* (AF=0.2% in Orkney; but observed as a singleton among Shetlander participants). Among the VIPs not yet incorporated into the PharmVar database, we observed a combined total of 7 potentially novel star alleles in *GSTM1* and *GSTT1*. The most common of these haplotypes included: *A+rs147668562~6329A>G(N85S) in *GSTM1*, especially among Orcadians (AF=1.6%) (Table S4).

**Fig. 3:**
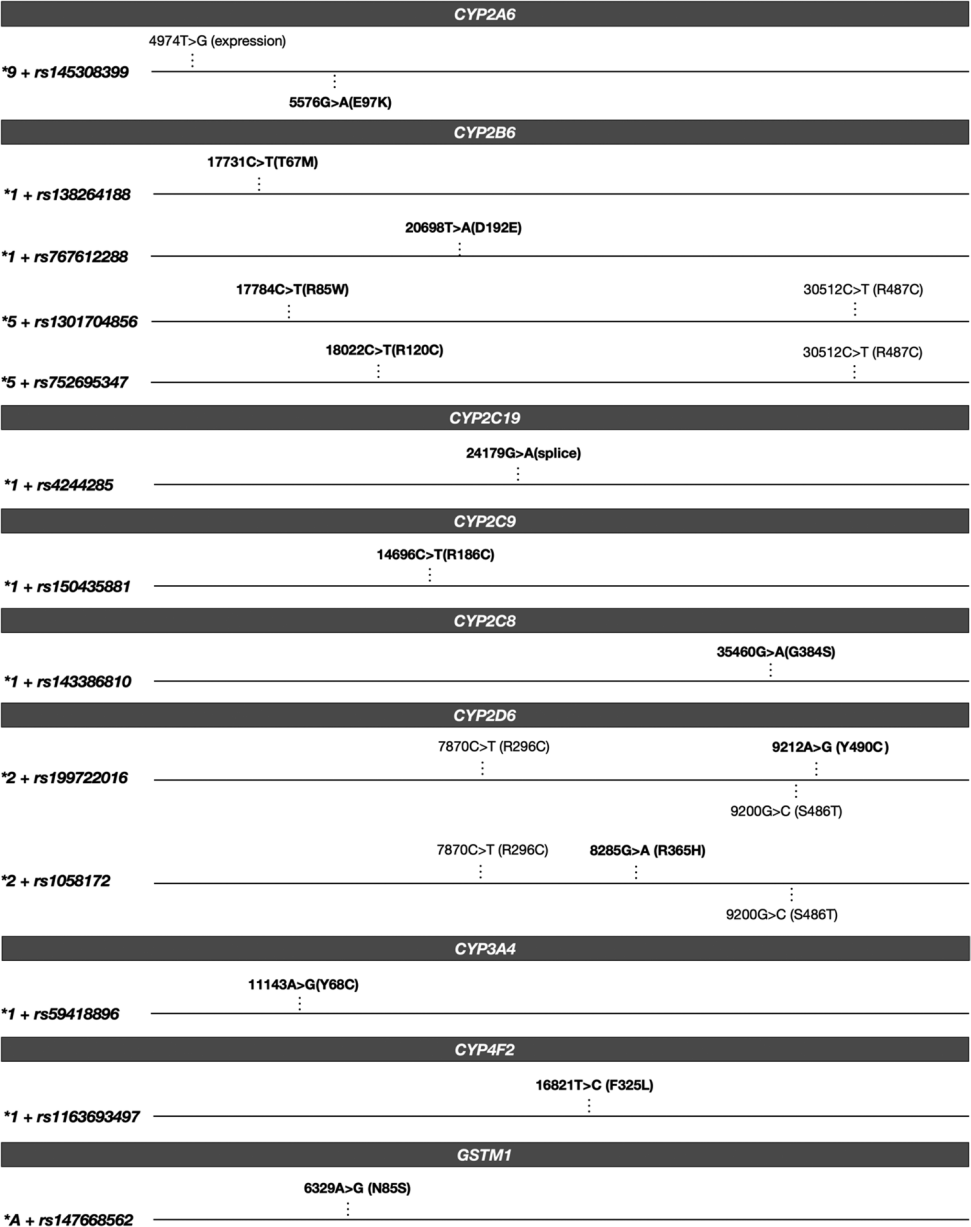
Depiction of selected potentially novel star alleles identified across Orcadian and Shetlander populations in the study. The variants in bold font-weight correspond to the indicated rsIDs. The variants with normal formatting, where present, correspond to the known/existing ‘backbone’ star alleles.

Only 13.2% (7 of 53) of the potential novel star alleles occurred in both Shetland and Orkney populations, 45.3% (24 of 53) occurred only in Shetlanders, and 41.5% (22 of 53) occurred only in Orcadians (Table S4). Most of these potentially novel alleles were absent from 1000G European populations, except *9+rs145308399~5576G>A(E97K) in *CYP2A6* and *2+rs150216909~7969C>T(R329C) in *CYP2D6*, both of which were singletons in the 1000G European dataset. Comparatively, CYP2A6*9+rs145308399~5576G>A(E97K), as aforementioned, was relatively more frequent in Orkney. Selected potentially novel star alleles defined by variants predicted to be deleterious are depicted in Figure 3 (functionality predictions by VEP plugins are provided in Table S3).

## Discussion

Geographically isolated populations tend to have unique genetic variation patterns characterised by founder variants or enrichment of otherwise rare variants. Several enriched rare deleterious and/or medically relevant variants have been observed in Shetland and Orkney as part of the Viking Genes project, owing to various forms of genetic drift^14–16^. Given that VIPs such as cytochrome P450 genes, among others, tend to harbour a plethora of genetic variants, this study assessed the PGx landscape in Shetland and Orkney in comparison with PGx variation across European populations represented in the 1000 Genomes Project, and data aggregated from literature curated by ClinPGx.

Overall, the distributions of the known pharmacogene variants and star alleles do not vary significantly across Shetland and Orkney. The frequencies of most clinically actionable alleles are comparable to frequencies observed in European cohorts included in this study, and those analysed in other studies^39,40^. Across genes with a star allele nomenclature, we highlight the occurrence of 53 potentially novel star alleles in Shetland and Orkney combined, majority of which are absent from the 1000G European dataset, and they haven’t been reported in studies curated by ClinPGx. Furthermore, majority of the potentially novel star alleles identified in the Shetland dataset were absent from Orkney and vice versa, thus exemplifying the value of population-specific PGx analysis. In some cases, it is possible to infer the age of the novel star allele from the inheritance pattern across deep pedigrees, e.g. three of the four carriers of *CYP2D6* rs199722016-9212A>G (Y490C) trace back to a common ancestor born in 1670, whereas both carriers of *CYP2C19* rs144036596-85273G>A (D360N) descend from a couple born in the 1770 s, also in Shetland. The *CYP2B6* rs138264188-17731C>T (T67M) variant is observed 18 times in our Orkney dataset and traces to multiple ancestors ~250 years ago, having originated before the 1760s. We predict that larger surveys will pick up more copies of these founder variants.

Predictions based on standardised diplotype-phenotype translation recommendations and CPIC guidelines (https://cpicpgx.org/guidelines) for key gene-drug pairs such as *CYP2B6* (efavirenz and sertraline), *CYP2C19* (clopidogrel and antidepressants), *CYP2C9* (warfarin and NSAIDs), *CYP2D6* (opioids, antidepressants, and tamoxifen), *IFNL3* (PEGIFN-α & RBV therapy), *SLCO1B1* (statins), and *VKORC1* (warfarin) showed that up to about 50% of the study participants in both Orkney and Shetland have phenotypes that might warrant treatment adjustments informed by (pre-emptive) pharmacogenetic testing. Moreover, every participant included in the study carried at least one clinically actionable PGx variant. Although our phenotype predictions were not compared with clinical drug response data from the participants, our findings suggest the need for evaluating the benefit of clinical PGx implementation to Shetlanders and Orcadians, especially considering the evidence of ADR reduction (30%) from other European initiatives such as the Pre-emptive Pharmacogenomic Testing for Preventing Adverse Drug Reactions (PREPARE) study^7^. Optimising drug therapy based on PGx (especially pre-emptively) alongside other precision medicine facets has the potential to mitigate ADRs and enhance treatment efficacy, which may translate into reduced cost of treatment and reducing strain on health systems in the Isles.

This study had some limitations. Firstly, as described in the Methods, there was a discrepancy in the sequencing coverage for the Shetland WGS (30x) and the Orkney WGS (20x) datasets (and indeed for the sample size), which could have affected frequency estimates and novel allele discovery. However, there were very few ambiguous star allele calls (usually expected with low-coverage data) in the lower-coverage Orkney dataset. In addition, the largely similar variant or star allele distributions between the Orcadian and Shetland datasets, the 1000G European datasets, and the ClinPGx reported frequencies justify the use of StellarPGx on the WGS datasets. Secondly, the potential novel alleles identified in the study require further laboratory validation, which was out of the scope of the study. These novel alleles, especially singletons, should therefore be interpreted with caution. We note that in many cases novel alleles were observed to segregate in pedigrees, corroborating their genotype calls. Thirdly, although we presented PGx phenotype predictions based on the diplotypes called in the analysis, several factors (such as age, environment, drug-drug interactions, lifestyle, and microbiome, among others) which are known to impact drug response were not considered in this study. Furthermore, it is difficult to make direct associations regarding the predicted deleterious variants identified in the VIPs in this study with drug response phenotypes.

## Conclusion

Herein, we have described the PGx variation across a subset of Shetlander and Orcadian participants that are part of the Viking Genes study. While the PGx landscape and distribution of actionable phenotypes is largely similar to observations across other European populations, the occurrence of potential novel and/or functionally relevant PGx variants or haplotypes enriched within families in these population isolates may warrant tailoring of PGx testing panels. Overall, understanding the distributions of PGx variants, star alleles and phenotypes across Shetland, Orkney, and potentially the broad scope of the Scottish Isles, is informative for research on utility and cost-effectiveness of clinical PGx testing for improved safety and efficacy of drug therapy.

### Future directions

Given the relatively high number of potential novel star alleles identified in the Shetland and Orkney datasets included in this study, further (experimental) characterisation of these haplotypes is warranted to support their inclusion in the PharmVar database (https://www.pharmvar.org). We also plan to examine the utility of Viking Genes whole exome sequence data for characterisation of PGx variation across the VIPs included in this study as exome data may be more feasible to generate for purposes of clinical PGx implementation.

Another critical aspect following this analysis is developing practical frameworks for clinical PGx implementation across the Scottish Isles and for communicating PGx test results to various healthcare stakeholders for the optimisation of patient care. This follows similar efforts emerging as part of the Viking Genes study^17^.

## Supplementary Information

Supplementary Information 1.

Supplementary Information 2.

Supplementary Information 3.

Supplementary Information 4.

Supplementary Information 5.

## Data Availability

The whole genome sequence for Orkney and Shetland are available from the European Genome-Phenome Archive, under accessions EGAD00001005194 and EGAD00001005378, respectively. Other research data and/or DNA samples from the VIKING I and ORCADES studies are available from viking@ed.ac.uk, following approval by the Viking Genes Data Access Committee and in line with the consent given by participants. There is neither Research Ethics Committee approval, nor consent from individual participants, to permit open release of the individual level research data underlying this study. The datasets analysed during the current study are therefore not publicly available. Instead, each approved project is subject to a data or materials transfer agreement (D/MTA) or commercial contract. The 1000 Genomes European datasets were downloaded from http://ftp.sra.ebi.ac.uk. Star allele frequencies for the broader European biogeographical group were obtained from ClinPGx (https://www.clinpgx.org/page/pgxGeneRef).

## Code availability

The description of how to run the StellarPGx pipeline can be found at https://github.com/SBIMB/StellarPGx.
