# Supplementary material for: Characterisation of pharmacogenomic variation in the Shetland and Orkney Isles in Scotland
Source: Sci Rep. 2025 Nov 26;15:42240. doi: 10.1038/s41598-025-26258-9 (PMC12658080; doi:10.1038/s41598-025-26258-9)
Supplement: Supplementary file 1 — Supplementary Information 1. [file 41598_2025_26258_MOESM1_ESM.pdf]

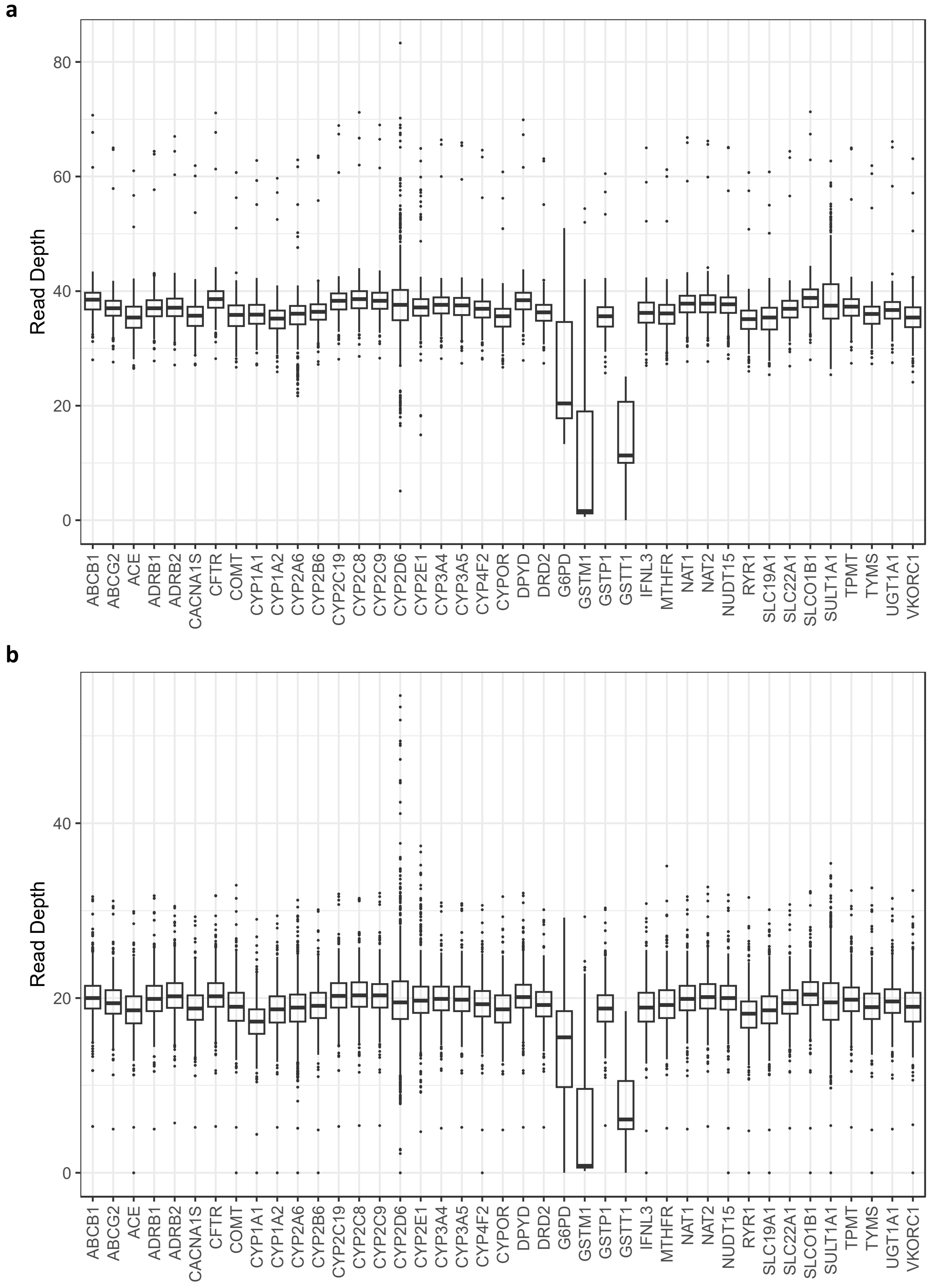

Supplementary Figure 1: Read depth distribution across the 41 pharmacogenes in the study based on whole genome sequence (WGS) data. (**a**) Read depth distribution based on WGS of the Shetland study population (n=498). (**b**) Read depth distribution based on WGS of the Orkney study population (n=1360). 12 Orcadian participants who are part of the 1000 Genomes European population high coverage (30x) dataset, but not part of the Viking Genes datasets, were later added to the study to bring the total of Orcadian participants to 1372. Average read depths for *G6PD* (X-linked), *GSTM1* (common gene deletion), and *GSTT1* (common gene deletion) were expectedly lower than those for other genes.
