# Supplementary material for: Characterisation of pharmacogenomic variation in the Shetland and Orkney Isles in Scotland
Source: Sci Rep. 2025 Nov 26;15:42240. doi: 10.1038/s41598-025-26258-9 (PMC12658080; doi:10.1038/s41598-025-26258-9)
Supplement: Supplementary file 2 — Supplementary Information 2. [file 41598_2025_26258_MOESM2_ESM.docx]

**Table S1:** Pharmacogenetic phenotype distributions across Shetland and Orkney populations compared with European populations represented in the 1000 Genomes Project (1000G) and the ClinPGx reference materials.

| **Genes with CPIC guidelines** | **CPIC clinical function / phenotype** | **Frequencies (%)** | | | |
| --- | --- | --- | --- | --- | --- |
|  |  | **Shetland** | **Orkney** | **European (1000G)** | **European**  **(ClinPGx)** |
| ***ABCG2*** | Normal function | 80.9 | 84.8 | 82.9 | 80.4 |
|  | Decreased function | 17.7 | 14.6 | 16.1 | 18.6 |
|  | Poor function | 1.4 | 0.6 | 1.0 | 1.1 |
| ***CFTR*** | Normal function | 95.8 | 95.1 | 97.7 | n/a |
|  | Actionable | 4.2 | 4.9 | 2.3 | n/a |
| ***CYP2B6*** | Normal metaboliser | 49.0 | 50.7 | 50.1 | 43.0 |
|  | Intermediate metaboliser | 34.3 | 32.7 | 33.2 | 38.0 |
|  | Poor metaboliser | 4.8 | 5.2 | 5.4 | 7.4 |
|  | Rapid metaboliser / ultrarapid metaboliser | 4.8 | 2.3 | 5.2 | 5.5 |
|  | Indeterminate | 7.0 | 9.1 | 6.1 | 6.1 |
| ***CYP2C19*** | Normal metaboliser | 44.0 | 45.0 | 36.5 | 39.6 |
|  | Intermediate metaboliser | 23.7 | 21.4 | 28.0 | 26.2 |
|  | Poor metaboliser | 2.0 | 0.9 | 1.3 | 2.4 |
|  | Rapid metaboliser | 24.7 | 27.4 | 28.6 | 27.1 |
|  | Ultrarapid metaboliser | 4.8 | 4.7 | 4.6 | 4.6 |
|  | Indeterminate | 0.8 | 0.6 | 1.0 | 0 |
| ***CYP2C9*** | Normal metaboliser | 67.5 | 71.2 | 62.2 | 62.8 |
|  | Intermediate metaboliser | 29.3 | 26.7 | 35.5 | 34.4 |
|  | Poor metaboliser | 2.8 | 2.0 | 2.3 | 2.6 |
|  | Indeterminate | 0.4 | 0.1 | 0.0 | <0.1 |
| ***CYP2D6*** | Normal metaboliser | 45.6 | 46.7 | 47.6 | 48.7 |
|  | Intermediate metaboliser | 38.8 | 36.7 | 37.8 | 38.3 |
|  | Poor metaboliser | 7.8 | 8.6 | 6.1 | 6.5 |
|  | Ultrarapid metaboliser | 1.0 | 1.4 | 3.1 | 2.8 |
|  | Indeterminate | 6.8 | 6.6 | 5.4 | 3.7 |
| ***CYP3A5*** | Expressor | 7.0 | 17.9 | 9.4 | 14.2 |
|  | Non-expressor | 93.0 | 82.1 | 90.6 | 85.7 |
| ***DPYD*** | Normal metaboliser | 94.0 | 93.4 | 93.9 | n/a |
|  | Intermediate metaboliser | 6.0 | 6.6 | 6.1 | n/a |
| ***IFNL3*** | Favourable response | 51.6 | 48.7 | 47.8 | n/a |
|  | Unfavourable response | 48.4 | 51.3 | 52.2 | n/a |
| ***NUDT15*** | Normal metaboliser | 96.4 | 98.6 | 99.6 | 98.6 |
|  | Intermediate metaboliser | 0.8 | 0.2 | 0.4 | 0.8 |
|  | Indeterminate | 2.8 | 1.2 | 0 | 0.6 |
| ***TPMT*** | Normal metaboliser | 91.6 | 90.8 | 92.1 | 90.9 |
|  | Intermediate metaboliser | 7.8 | 8.9 | 6.7 | 8.4 |
|  | Poor metaboliser | 0.4 | 0.1 | 0 | 0.2 |
|  | Indeterminate | 0.2 | 0.2 | 1.3 | 0.5 |
| ***SLCO1B1*** | Normal function | 60.4 | 60.5 | 66.6 | 65.6 |
|  | Decreased function | 23.3 | 20.9 | 28.0 | 28.2 |
|  | Poor function | 1.6 | 2.1 | 1.9 | 2.9 |
|  | Increased function | 4.4 | 4.8 | 2.5 | 3.0 |
|  | Indeterminate | 10.2 | 11.7 | 1.0 | 0.1 |
| ***UGT1A1*** | Normal metaboliser | 41.6 | 49.0 | 45.1 | n/a |
|  | Intermediate metaboliser | 41.8 | 42.1 | 43.0 | n/a |
|  | Poor metaboliser | 15.1 | 7.6 | 8.8 | n/a |
|  | Indeterminate | 1.6 | 1.3 | 3.1 | n/a |
| ***VKORC1*** | Normal warfarin dose | 38.4 | 40.4 | 37.8 | n/a |
|  | Possibly decreased warfarin dose | 48.4 | 45.9 | 45.7 | n/a |
|  | Decreased warfarin dose | 13.3 | 13.8 | 16.5 | n/a |

CPIC **–** Clinical Pharmacogenetics Implementation Consortium
