# Supplementary material for: Characterisation of pharmacogenomic variation in the Shetland and Orkney Isles in Scotland
Source: Sci Rep. 2025 Nov 26;15:42240. doi: 10.1038/s41598-025-26258-9 (PMC12658080; doi:10.1038/s41598-025-26258-9)
Supplement: Supplementary file 3 — Supplementary Information 3. [file 41598_2025_26258_MOESM3_ESM.docx]

**Table S2:** Distribution of rare star alleles across cytochrome P450 genes analysed in the study. Star alleles show here have an allele frequency <1% in Shetland and Orkney.

| Star alleles | Defining variants | CPIC/known clinical function | Frequencies (%) | | | |
| --- | --- | --- | --- | --- | --- | --- |
|  |  |  | **Shetland** | **Orkney** | **European (1000G)** | **European**  **(ClinPGx)^a^** |
| *CYP2A6* | | | | | | |
| **1x2* | Duplication of **1* | Increased mRNA expression | 0.3 | 0.3 | 0.9 | n/a |
| **4* | Gene deletion | no mRNA expression | 0.6 | 0.4 | 0.9 | n/a |
| **5* | rs5031017 (G479V),  3’-UTR conversion (expression) | Decreased enzyme activity | 0.2 | <0.1 | 0 | n/a |
| **7* | rs5031016 (I471T),  3’-UTR conversion (expression) | Decreased enzyme activity | 0.1 | 0.1 | 0.2 | n/a |
| **12* | CYP2A7-2A6 hybrid (exons 1-2 from CYP2A7) |  | 0 | <0.1 | 0 | n/a |
| **42* | rs763469584 (I268T) |  | 0 | <0.1 | 0 | n/a |
| **47* | *CYP2A7-2A6* hybrid (exons 1-8 from *CYP2A7*) | Non-functional | 0.5 | <0.1 | 0 | n/a |
| *CYP2B6* | | | | | | |
| **3* | rs45482602 (S259R) | Uncertain | 0 | 0 | 0.1 | 0.2 |
| **8* | rs12721655 (K139E) | No function | 0.1 | 0.2 | 0 |  |
| **10* | rs34883432 (Q21L), rs8192709 (R22C) | Uncertain | 0 | 0.6 | 0.6 |  |
| **13* | rs12721655 (K139E), rs3745274 (Q172H, splice defect), rs2279343 (K262R) | No function | 0 | 0 | 0.1 |  |
| **14* | rs35773040 (R140Q) | Uncertain | 0 | <0.1 | 0 | 0.5 |
| **36* | rs34223104 (expression), rs3745274 (Q172H, splice defect), rs2279343 (K262R) | Decreased | 0.4 | 0 | 0 |  |
| **45* | rs58871670 (V183I) | Uncertain | 0.2 | 0.1 | 0 |  |
| *CYP2C19* | | | | | | |
| **4* | rs28399504 (M1V), rs3758581 (I331V) | No function | 0.3 | 0.3 | 0.1 | 0.2 |
| **8* | rs41291556 (W120R), rs3758581 (I331V) | No function | 0.3 | 0.1 | 0.3 | 0.3 |
| **11* | rs58973490 (R150H), rs3758581 (I331V) | Normal | 0.4 | 0.8 | 0.2 |  |
| **35* | rs12769205 (splice defect), rs3758581 (I331V) | No function | 0 | 0.2 | 0 | 0 |
| **36* | *CYP2C19* full gene deletion | No function | 0 | 0 | 0.1 |  |
| *CYP2C9* | | | | | | |
| **8* | rs7900194 (R150H) | Decreased | 0 | 0 | 0.2 | 0.2 |
| **9* | rs2256871 (H251R) | Normal | 0 | 0 | 0.1 |  |
| **11* | rs28371685 (R335W) | Decreased | 0.6 | 0.2 | 0.2 | 0.2 |
| **12* | rs9332239 (P489S) | Decreased | 0.1 | <0.1 | 0.3 |  |
| **29* | rs182132442 (P279T) | Decreased | 0 | 0 | 0.1 |  |
| **39* | rs762239445 (G98V) | No function | 0 | 0.1 | 0 | 0 |
| **45* | rs199523631 (R132W) | No function | 0 | 0 | 0.1 | <0.1 |
| **73* | rs17847037 (R150C) | Unknown | 0 | <0.1 | 0 | <0.1 |
| *CYP2C8* | | | | | | |
| **2* | rs11572103 (I269F) | Uncertain | 0 | 0 | 0.4 |  |
| *CYP2D6* | | | | | | |
| **1x2* | Duplication of *1 | Increased | 0.1 | 0.4 | 0.5 | 0.8 |
| **13+*1* | Hybrid tandem arrangement | Normal | 0 | 0 | 0.1 | <0.1 |
| **13+*2* | Hybrid tandem arrangement | Normal | 0 | 0.4 | 0.2 |  |
| **3x2* | Duplication of **3* | No function | 0.1 | 0 | 0 | <0.1 |
| **9x2* | Duplication of **9* | Normal | 0 | 0 | 0.1 | <0.1 |
| **13* | *CYP2D7-2D6* hybrid | No function | 0 | 0 | 0.2 | <0.1 |
| **15* | rs774671100 (frameshift) | No function | 0 | 0.3 | 0 | <0.1 |
| **17* | rs28371706 (T107I), rs16947 (R296C), rs1135840 (S486T) | Decreased | 0 | <0.1 | 0.2 | 0.4 |
| **22* | rs138100349 (R28C) | Uncertain | 0 | 0 | 0.3 | 0.3 |
| **27* | rs769157652 (E410K) | Normal | 0 | 0.1 | 0 | 0 |
| **34* | rs16947 (R296C) | Normal | 0.1 | 0.5 | 0 | 1.1 |
| **39* | rs1135840 (S486T) | Normal | 0.2 | 0.8 | 0.1 | 1.4 |
| **45* | rs28371710 (E155K), rs16947 (R296C), rs1135840 (S486T) | Normal | 0 | <0.1 | 0 | 0 |
| **31* | rs16947 (R296C), rs267608319 (R440H), rs1135840 (S486T) | No function | 0 | 0 | 0.2 | 0.1 |
| **32* | rs16947 (R296C), rs28371725 (splicing defect), rs769157652 (E410K), rs1135840 (S486T) | Uncertain | 0.1 | <0.1 | 0.3 | 0.3 |
| **35x2* | Duplication of *35 | Increased | 0.1 | 0 | 0 | <0.1 |
| **36* | Exon 9 conversion to *CYP2D7* | No function | 0 | <0.1 | 0 | <0.1 |
| **36+*10* | Hybrid tandem arrangement | Decreased | 0 | <0.1 | 0 |  |
| **43* | rs28371696 (R26H) | Uncertain | 0 | 0 | 0.1 | <0.1 |
| **65* | rs1065852 (P34S), rs16947 (R296C), rs1135840 (S486T) | No function | 0 | 0.1 | 0 |  |
| **69* | rs1065852 (P34S), rs16947 (R296C), rs28371725 (splice defect), rs1135840 (S486T) | No function | 0 | 0.1 | 0.1 | 0.1 |
| **74* | rs28371703 (L91M) | Uncertain | 0 | <0.1 | 0 | 0 |
| **83* | Exon 9 conversion to *CYP2D7*, rs1135840 (S486T) | Uncertain | 0 | 0.1 | 0 |  |
| **108* | rs61736517 (H352R), rs202102799 (Y355C) | Unknown | 0.7 | 0.1 | 0.3 | 0.3 |
| **117* | rs16947 (R296C), rs78209835 (D337N), rs1135840 (S486T) | Unknown | 0 | 0 | 0.4 | 0.4 |
| **119* | rs28371725 (splicing defect) | Decreased | 0.1 | 0.1 | 0 | 0 |
| **122* | rs61745683 (V370I) | Unknown | 0 | <0.1 | 0 | 0 |
| **139* | rs1058172 (R365H) | Unknown | 0.7 | 0.6 | 0 | 0 |
| **160* | rs28371703 (L91M), rs28371704 (H94R), rs16947 (R296C), rs1135840 (S486T) | Unknown | 0.1 | 0 | 0 |  |
| *CYP3A4* | | | | | | |
| **2* | rs55785340 (S222P) | Uncertain | 0.0 | 0.0 | 0.2 | n/a |
| **7* | rs56324128 (G56D) | Uncertain | 0.0 | 0.0 | 0.2 | n/a |
| **8* | rs72552799 (R130Q) | Uncertain | 0.0 | 0.0 | 0.1 | n/a |
| **11* | rs67784355 (T363M) | Uncertain | 0.0 | 0.0 | 0.1 | n/a |
| **15* | rs4986907 (R162Q) | Uncertain | 0.0 | 0.0 | 0.1 | n/a |
| **23* | rs57409622 (R162W) | Uncertain | 0.0 | <0.1 | 0.0 | n/a |
| **37* | rs35599367 (splice defect), rs4986910 (M445T) | Uncertain | 0.0 | <0.1 | 0.0 | n/a |
| *CYP3A5* | | | | | | |
| **6* | rs10264272 (splice defect) | No function | 0 | 0 | 0.3 | 0.2 |
| *CYP4F2* | | | | | | |
| **2* | rs3093105 (W12G) | Unknown | 0.3 | 0.6 | 0 | 16^b^ |
| **17* | rs4020346 (T472A) | Unknown | 0 | 0.2 | 0.5 |  |

^a^ Number of participants in ClinPGx aggregate analyses vary (*CYP2B6*: n=69572, *CYP2C19*: n=71782, *CYP2C9*: n≈91200, *CYP2D6*: n=65090, *CYP3A5*: n=5607, *CYP4F2*: n=77524).

^b^ The differences in allele frequencies of *CYP4F2*2*, **3*, and **4* are due to differences in phasing approaches for the core variants rs3093105 (W12G) and rs2108622 (V433M).
