# Supplementary material for: Characterisation of pharmacogenomic variation in the Shetland and Orkney Isles in Scotland
Source: Sci Rep. 2025 Nov 26;15:42240. doi: 10.1038/s41598-025-26258-9 (PMC12658080; doi:10.1038/s41598-025-26258-9)
Supplement: Supplementary file 5 — Supplementary Information 5. [file 41598_2025_26258_MOESM5_ESM.docx]

**Table S4:** Potentially novel pharmacogene major star alleles and suballeles observed in Shetland and Orkney datasets. The definitions of existing/backbone alleles are provided in **Table 1** and **Table 2**. Variants with deleterious variant effect predictions (consensus of SIFT, Polyphen-2, LRT, MutationAssessor, PROVEAN, CADD, and AlphaMissense) are written in red text. Frameshifts, stop gain variants, and splice defects were considered deleterious by default. This table includes only pharmacogenes with an existing star allele nomenclature (see **Table S3** for information on all pharmacogenetic variants identified in the study).

| **#** | **Diplotype and variant details** | **Count for novel haplotype(s)** | |
| --- | --- | --- | --- |
|  |  | **Shetland** | **Orkney** |
| ***CYP2A6*** | | | |
| 1 | *1/[*46 + no_rsID~6772G>A (G144D)] | 3 | 0 |
|  | *46/[*46 + no_rsID~6772G>A (G144D)] |  |  |
| 2 | *1/[*46 + rs143841823~10048T>C (M352T)] | 5 | 0 |
|  | *46/[*46 + rs143841823~10048T>C (M352T)] |  |  |
| 3 | *1/[*1 + rs148693084~9418T>C (I300T)] | 1 | 0 |
| 4 | *2/[*35 + rs28399433~4974T>G (*9; expression)] | 3 | 0 |
|  | *4/[*35 + rs28399433~4974T>G (*9; expression)] |  |  |
| 5 | *1/[*9 + rs145157460~8511C>G (R257G)] | 4 | 2 |
|  | *9/[*9 + rs145157460~8511C>G (R257G)] |  |  |
|  | *18/[*9 + rs145157460~8511C>G (R257G)] |  |  |
|  | *35/[*9 + rs145157460~8511C>G (R257G)] |  |  |
| 6 | *1/[*1 + rs138978736~8457C>A (Q239K)] | 0 | 4 |
|  | *9/[*1 + rs138978736~8457C>A (Q239K)] |  |  |
| 7 | *1/[*9 + rs145308399~5576G>A (E97K)] | 0 | 10 |
|  | *9/[*9 + rs145308399~5576G>A (E97K)] |  |  |
|  | *21/[*9 + rs145308399~5576G>A (E97K)] |  |  |
| ***CYP2B6*** | | | |
| 1 | *1/[*5 + rs1301704856~17784C>T (R85W)] | 5 | **0** |
|  | *2/[*5 + rs1301704856~17784C>T (R85W)] |  |  |
|  | *5/[*5 + rs1301704856~17784C>T (R85W)] |  |  |
| 2 | *1/[*5 + rs752695347~18022C>T (R120C)] | 5 | **0** |
|  | *5/[*5 + rs752695347~18022C>T (R120C)] |  |  |
|  | *9/[*5 + rs752695347~18022C>T (R120C)] |  |  |
| 3 | *1/[*1 + rs138264188~17731C>T (T67M)] | 1 | 18 |
|  | *2/[*1 + rs138264188~17731C>T (T67M)] |  |  |
|  | *5/[*1 + rs138264188~17731C>T (T67M)] |  |  |
|  | *6/[*1 + rs138264188~17731C>T (T67M)] |  |  |
| 4 | *2/[*6 + rs8192709~5071C>T (R22C)] | 2 | 0 |
|  | *6/[*6 + rs8192709~5071C>T (R22C)] |  |  |
| 5 | *1/[*1 + rs17411404~26491C>A (T423N)] | 0 | 4 |
|  | *6/[*1 + rs17411404~26491C>A (T423N)] |  |  |
| 6 | *1/[*1 + rs183040778~18136C>T (R158W)] | 0 | 3 |
|  | *5/[*1 + rs183040778~18136C>T (R158W)] |  |  |
| 7 | *4/[*1 + rs767612288~20698T>A (D192E)] | 0 | 5 |
|  | *7/[*1 + rs767612288~20698T>A (D192E)] |  |  |
|  | *9/[*1 + rs767612288~20698T>A (D192E)] |  |  |
| 8 | *1/[*1 + rs770007043~23071G>A (D266N)] | 0 | 1 |
| 9 | [*1/*2] + rs749817915~5132A>T (N42I) | 0 | 1 |
| ***CYP2C19*** | | | |
| 1 | [*2/*17] + rs183701923~22893C>T (R186C) | 1 | 0 |
| 2 | *1/[*1 + rs144036596~ 85273G>A (D360N)] | 2 | 0 |
| 3 | *17/[*1 + rs142974781~17826C>T (R150C)] | 0 | 1 |
| 4 | [*1 + rs17878459~17485G>C (E92D)]/[*1 + rs4244285~24179G>A (splice)] | 0 | 1 |
| 5 | *1/[*1 + rs4244285~24179G>A (splice)] | 0 | 7 |
|  | *11/[*1 + rs4244285~24179G>A (splice)] |  |  |
| ***CYP2C19* potentially novel suballeles** | | | |
| 1 | *2/[*2 + rs58973490~17827G>A (R150H)] | 1 | 6 |
|  | *17/[*2 + rs58973490~17827G>A (R150H)] |  |  |
|  | *38/[*2 + rs58973490~17827G>A (R150H)] |  |  |
| ***CYP2C9*** | | | |
| 1 | *1/[*1 + rs150435881~14696C>T (R186C)] | 1 | 0 |
| 2 | *1/[*1 + rs1295294399~16032A>C (S242R)] | 1 | 0 |
| ***CYP2C8*** | | | |
| 1 | [*1/*4] + rs576554998~9553C>T (P166L) | 1 | 0 |
| 2 | *1/[*1 + rs11572080~7225G>A (R139K)] | 1 | 6 |
|  | *4/[*1 + rs11572080~7225G>A (R139K)] |  |  |
| 3 | *1/[*1 + rs143386810~35460G>A (G384S)] | 1 | 0 |
| 4 | *1/*1 + rs10509681~35506A>G (K399R) | 0 | 3 |
| ***CYP2D6*** | | | |
| 1 | [*1/*59] + rs199609589~7007G>A (stop-gained) | 1 | 0 |
| 2 | *2/[*2 + rs199722016~9212A>G (Y490C)] | 2 | 0 |
|  | *4/[*2 + rs199722016~9212A>G (Y490C)] | 2 | 0 |
| 3 | *5/[*39 + rs1058172~8285G>A (R365H)] | 1 | 1 |
|  | *10/[*39 + rs1058172~8285G>A (R365H)] |  |  |
| 4 | *1/[*2 + rs150216909~7969C>T (R329C)] | 1 | 3 |
|  | [*2 + rs150216909~7969C>T (R329C)] / [*2 + rs150216909~7969C>T (R329C)] |  |  |
| 5 | *4/[*59 + rs1065852~5119C>T (P34S)] | 0 | 1 |
| 6 | *5/[*41 + rs61745683~8299G>A (V370I) + rs2146934648~7539A>C (T249P)] | 0 | 1 |
| 7 | *2/[*2 + rs745365204~6963G>A (R201H)] | 0 | 1 |
| 8 | *2/[*35 + rs745365204~6963G>A (R201H)] | 0 | 5 |
|  | *4/[*35 + rs745365204~6963G>A (R201H)] |  |  |
|  | *9/[*35 + rs745365204~6963G>A (R201H)] |  |  |
|  | *35/[*35 + rs745365204~6963G>A (R201H)] |  |  |
| 9 | *5/[*2 + rs1058172~8285G>A (R365H)] | 0 | 2 |
|  | *10/[*2 + rs1058172~8285G>A (R365H)] |  |  |
| 10 | *2/[*35 + rs769157652~8873G>A (E410K)] | 0 | 1 |
| 11 | [*1/*2] + rs72549358~5038G>A (V7M) | 0 | 1 |
| 12 | [*10/*2] + rs28371703~5992C>A (L91M) | 0 | 1 |
| 13 | [*1/*10] + rs28371704~6002A>G (H94R) | 0 | 1 |
| ***CYP2D6* potential novel suballeles** | | | |
| 1 | *4/*4[suballele-with-rs3915951~8177G>T (R329L)] | 0 | 1 |
| 2 | *39/*4[suballele-with-rs759631541~9121C>G (L460V)-CN=3] | 0 | 1 |
| ***CYP3A4*** | | | |
| 1 | *1/[*1 + rs59418896~11143A>G (Y68C)] | 4 | 0 |
| 2 | [*10/*3] + rs35599367~20493C>T (splicing defect) | 1 | 0 |
| ***CYP3A5 potential novel suballeles***  *CYP3A5*3* is a non-functional star allele due to the occurrence of rs776746-C (splice defect).  Therefore the suballeles presented here are also non-functional | | | |
| 1 | *3/[**3*; in *cis* with rs41279857~12400C>A (S100C)] | 3 | 0 |
| 2 | *3/[*3; in *cis* with rs1245832664~32278A>G (E362G)] | 1 | 0 |
| 3 | *3/[*3; in *cis* with rs28365092~32304A>G (I371V)] | 1 | 0 |
| 4 | *3/[*3; in *cis* with rs28371766~5178G>A (splice donor)] | 1 | 0 |
| 5 | *3/[*3; in *cis* with rs201260783~20979T>A (stop-gained)] | 1 | 0 |
| ***CYP4F2*** | | | |
| 1 | *1/[*1 + rs1163693497~16821T>C (F325L)] | 2 |  |
|  | *3/[*1 + rs1163693497~16821T>C (F325L)] |  |  |
| 2 | *1/[*3 + rs150579280~7524A>C (S100R)] | 4 | 0 |
| 3 | *4/[*3 + rs150579280~7524A>C (S100R)] | 0 | 1 |
| 4 | *4/[*1 + rs4020346~24155A>G (T472A)] | 2 | 0 |
|  | *1/[*1 + rs4020346~24155A>G (T472A)] |  |  |
| ***GSTM1*** | | | |
| *1* | **A / [*A + rs147668562~6329A>G (N85S)]* | 2 | 43 |
|  | **B /[*A + rs147668562~6329A>G (N85S)]* |  |  |
|  | **0 / [*A + rs147668562~6329A>G (N85S)]* |  |  |
| *2* | **0 / [*A + rs147668562~6329A>G (N85S) + rs1570638172~6431T>G (C87W)]* | 0 | 1 |
| 3 | *[*A + rs449856 (S210T)]/[*A + rs449856 (S210T)]* | 2 | 0 |
| 4 | **B / *B* + rs142484086 (R145W) | 1 | 0 |
| 5 | *[*B + rs1570638172 (C87W)] / [*B + rs1570638172 (C87W)]* | 2 | 0 |
|  |  |  |  |
| ***GSTT1*** | | | |
| *1* | *0 / [**A* + rs1601976243 (T104P)] | 1 | 1 |
| *2* | *0 / [**A* + rs1601976060 (stop-gained)] | 1 | 0 |
